# Incidence and survival analyses for occult lung cancer between 2004 and 2015: a population-based study

**DOI:** 10.1186/s12885-021-08741-4

**Published:** 2021-09-09

**Authors:** Lei-Lei Wu, Chong-Wu Li, Wei-Kang Lin, Li-Hong Qiu, Dong Xie

**Affiliations:** 1 Department of Thoracic Surgery, Shanghai Pulmonary Hospital, School of Medicine, Tongji University, Shanghai, 200433 P. R. China; 2 Sun Yat-sen University Cancer Center, State Key Laboratory of Oncology in South China, Collaborative Innovation Center for Cancer Medicine, Guangzhou, 510060 P. R. China

**Keywords:** Occult lung cancer, Incidence, Survival, Surveillance, Epidemiology, And end results database

## Abstract

**Background:**

This study aimed to investigate the incidence and long-term survival outcomes of occult lung cancer between 2004 and 2015.

**Methods:**

A total of 2958 patients were diagnosed with occult lung cancer in the 305,054 patients with lung cancer. The entire cohort was used to calculate the crude incidence rate. Eligible 52,472 patients (T1-xN0M0, including 2353 occult lung cancers) were selected from the entire cohort to perform survival analyses after translating T classification according to the 8th TNM staging system. Cancer-specific survival curves for different T classifications were presented.

**Results:**

The crude incidence rate of occult lung cancer was 1.00 per 100 patients, and it was reduced between 2004 and 2015 [1.4 per 100 persons in 2004; 0.6 per 100 persons in 2015; adjusted risk ratio = 0.437, 95% confidence interval (CI) 0.363–0.527]. In the survival analysis, there were 2206 death events in the 2353 occult lung cancers. The results of the multivariable analysis revealed that the prognoses with occult lung cancer were similar to patients with stage T3N0M0 (adjusted hazard ratio = 1.054, 95% CI 0.986–1.127, *p* = 0.121). Adjusted survival curves presented the same results. In addition, adjusted for other confounders, female, age ≤ 72 years, surgical treatment, radiotherapy, adenocarcinoma, and non-squamous and non-adenocarcinoma non-small cell carcinoma were independent protective prognostic factors (all *p* < 0.05).

**Conclusions:**

Occult lung cancer was uncommon. However, the cancer-specific survival of occult lung cancer was poor, therefore, we should put the assessment of its prognoses on the agenda. Timely surgical treatment and radiotherapy could improve survival outcomes for those patients. Besides, we still need more research to confirm those findings.

## Background

Lung cancer still is the leading malignancy in the global cancer spectrum of morbidity and mortality [1]. Lung cancer mainly comprises non-small cell lung cancer (NSCLC) and small cell lung cancer (SCLC), with more than 83% of all cases being NSCLC [2]. Because of late diagnosis and tumor recurrence, the 5-year overall survival rate of patients with NSCLC and SCLC remains low (approximately 23 and 6%, respectively) [2, 3]. Tumor proven by the presence of malignant cells in sputum or bronchial washings but not visualized by imaging or bronchoscopy is considered as occult lung cancer [4, 5]. Previous studies on the incidence of occult lung cancer have only analyzed groups of stroke patients, or incidental case reports of other diseases [6–9]. Therefore, for lung cancer patients, the incidence information of occult lung cancer remains insufficient.

In addition, accurate tumor-lymph node-metastasis (TNM) staging means that the prognosis of the patients is accurate [10, 11]. Patients with stage IA (classification T1N0M0) have the best long-term survival outcomes in all lung cancers [10]. In the guidelines of the National Comprehensive Cancer Network, occult lung cancers are classified as TxN0M0 [12]. Thus, the prognosis of occult lung cancer patients remains unclear because of the unclear TNM classification. The prognoses of diseases have an effect on treatment selection and patients’ management. However, there was no data about the incidence rate and survival analyses of occult lung cancer in the previous studies. Thus, we aimed to investigate the incidence rate and prognostic level of those patients with occult lung cancer.

## Methods

### Patients

**Fig. 1:**
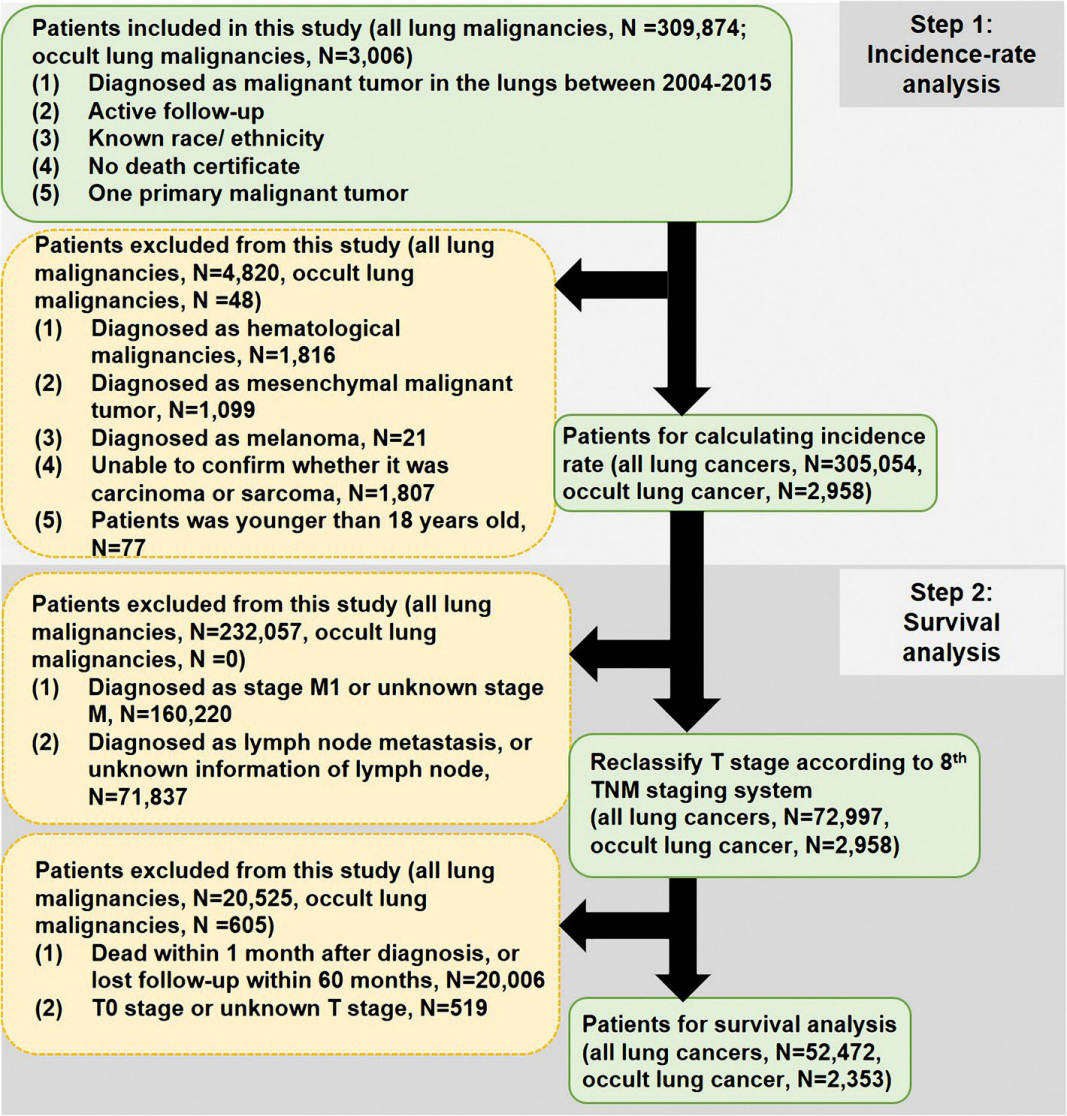
The flow chart of this study

The Ethics Committee of Shanghai Pulmonary Hospital approved this study and considered this study exempt from ethical review because existing data without patient identifiers were used. This study majorly included two parts, incidence-rate analysis (step 1) and survival analysis (step 2). We retrospectively recruited patients who were histologically diagnosed with malignant tumor in the lungs as their first primary malignancy from 2004 to 2015 in Surveillance, Epidemiology, and End Results (SEER) database, which contains clinicopathological and survival data of cancer patients from 18 registries. Therefore, the present study could be considered as a multi-center analysis. The selection criteria of patients were shown in Fig. 1. A total of 305,054 patients (including 2958 occult lung cancers) were used to perform incidence analysis after step-one case selection. Next, we processed step-two case selection. There were 52,472 eligible patients (including 2353 occult lung cancers) for survival analysis. The detailed information was presented as Fig. 1. All patient records were anonymized before analysis. Information collected from the SEER database included sex, race/ ethnicity, survival time, cause on disease, age at diagnosis, tumor size, approach of treatment (including surgical treatment, radiotherapy, and chemotherapy), tumor differentiation, histological subtype, tumor location, TNM stage, and marital status.

### Follow-up

Cancer-specific survival, which was the duration from the date of diagnosis to death caused by lung cancer, was regarded as our observational endpoint. For survival analysis, follow-up duration ranged from 1.0 to 155.0 months, with a median of 27.0 months. Those patients who entered the survival analysis had definitive survival status, death or alive.

### Statistical analysis

All statistical analysis was performed using SPSS statistics 25.0 software (IBM SPSS, Inc., Chicago, IL, USA), and GraphPad Prism 8 (https://www.graphpad.com/scientific-software/prism/). Risk ratios (RRs), hazard ratios (HRs) and 95% confidence intervals (CIs) were calculated using multivariable Logistic regression analysis and Cox regression analysis, respectively (regression method was Enter selection). The average value of each covariate was calculated by the multivariable Cox regression model, and estimated the adjusted survival curves of T classification. Statistical tests were considered statistically significant with two-sided *p* value < 0.05.

## Results

### Patient characteristics

In the step-one case selection, there were 305,054 patients (including 2958 occult lung cancers) for calculating incidence. Majority of the patients were male (*N* = 162,448, 53.3%), and 248,125 (81.3%) were non-Hispanic whites. The median age was 68 years old (range from 18 years to 104 years). The detailed information of patient characteristics was shown in Table 1.

**Table 1:** Clinical characteristic of lung cancer patients from Surveillance Epidemiology and End Results database

	***All patients*** ***(N = 305,054)***	
***Variables***	***N***	***%***
**Sex**		
Male	162,448	53.3
Female	142,606	46.7
**Race**		
Non-Hispanic whites	248,125	81.3
Non-Hispanic others	56,929	18.7
**Age (year)**		
≤ 68	159,281	52.2
> 68	145,773	47.8
Median (range)	68 (18–104)	
z **Grade**		
Well	16,073	5.3
Moderate	52,350	17.2
Poor	85,221	27.9
Undifferentiated	14,184	4.6
Unknown	13,7226	45.0
**Tumor Location**		
Main bronchus	15,819	5.2
Upper lobe	155,796	51.1
Middle lobe	13,314	4.4
Lower lobe	77,101	25.3
Overlapping lesion of lung	3973	1.2
Unknown	39,051	12.8
**Radiotherapy**		
No	174,025	57.0
Yes	128,452	42.2
Unknown	2577	0.8
**Chemotherapy**		
No	159,187	52.2
Yes	145,867	47.8
**Marital status**		
Married	156,432	51.3
Non-married	136,241	44.6
Unknown	12,381	4.1
**Surgical treatment**		
None	233,739	76.5
Limited resection	14,070	4.6
Lobectomy	51,453	16.8
Pneumonectomy	4030	1.4
Unknown surgical approach	729	0.2
Unknown	1543	0.5
**Year at diagnosis**		
2004	23,625	7.7
2005	22,774	7.6
2006	24,250	7.9
2007	25,094	8.2
2008	25,384	8.3
2009	25,936	8.5
2010	25,606	8.4
2011	25,631	8.4
2012	26,120	8.6
2013	26,344	8.6
2014	26,874	8.8
2015	27,416	9.0
**Occult lung cancer**		
Yes	2958	1.0
No	302,096	99.0

After step-two case selection, eligible 52,472 patients (including 2353 occult lung cancers) entered into processing of survival analysis. Male patients accounted for 51.2% (*N* = 26,858). Age at diagnosis ranged from 18 years old to 100 years old (median 70 years). The major part of histological subtypes belonged to adenocarcinoma (*N* = 23,406, 44.6%) as shown in Table 2.

**Table 2:** Clinicopathological characteristic of lung cancer patients for survival analysis

	***All patients*** ***(N = 52,472)***	
***Variables***	***N***	***%***
**Sex**		
Male	26,858	51.2
Female	25,614	48.8
**Race**		
Non-Hispanic whites	43,818	83.5
Non-Hispanic others	8654	16.5
**Age (year)**		
≤ 68	23,070	44.0
> 68	29,402	56.0
Median (range)	70 (18–100)	
**Grade**		
Well	5579	10.6
Moderate	16,142	30.8
Poor	16,855	32.1
Undifferentiated	1745	3.3
Unknown	12,151	23.2
**Tumor Location**		
Main bronchus	1271	2.4
Upper lobe	30,463	58.0
Middle lobe	2423	4.6
Lower lobe	15,734	30.0
Overlapping lesion of lung	607	1.2
Unknown	1974	3.8
**Radiotherapy**		
No	37,612	71.7
Yes	14,508	27.6
Unknown	352	0.7
**Chemotherapy**		
No	39,127	74.6
Yes	13,345	25.4
**Marital status**		
Married	27,089	51.6
Non-married	23,504	44.8
Unknown	1879	3.6
**Surgical treatment**		
None	22,136	42.2
Limited resection	5784	11.0
Lobectomy	23,228	44.2
Pneumonectomy	984	1.9
Unknown surgical approach	29	0.1
Unknown	311	0.6
**Histological subtypes**		
Squamous cell carcinoma	16,691	31.8
Adenocarcinoma	23,406	44.6
Non-squamous and non-adenocarcinoma NSCLC	5128	9.8
Small-cell carcinoma	2583	4.9
Unknown non-sarcoma carcinoma	477	0.9
Unknown NSCLC	4187	8.0
**T classification**		
T1a	1752	3.3
T1b	9439	18.0
T1c	8412	16.1
T2a	11,509	21.9
T2b	3784	7.2
T3	4180	8.0
T4	11,043	21.0
Tx (occult)	2353	4.5

*NSCLC* non-small cell lung cancer

### Incidence-rate analysis

**Fig. 2:**
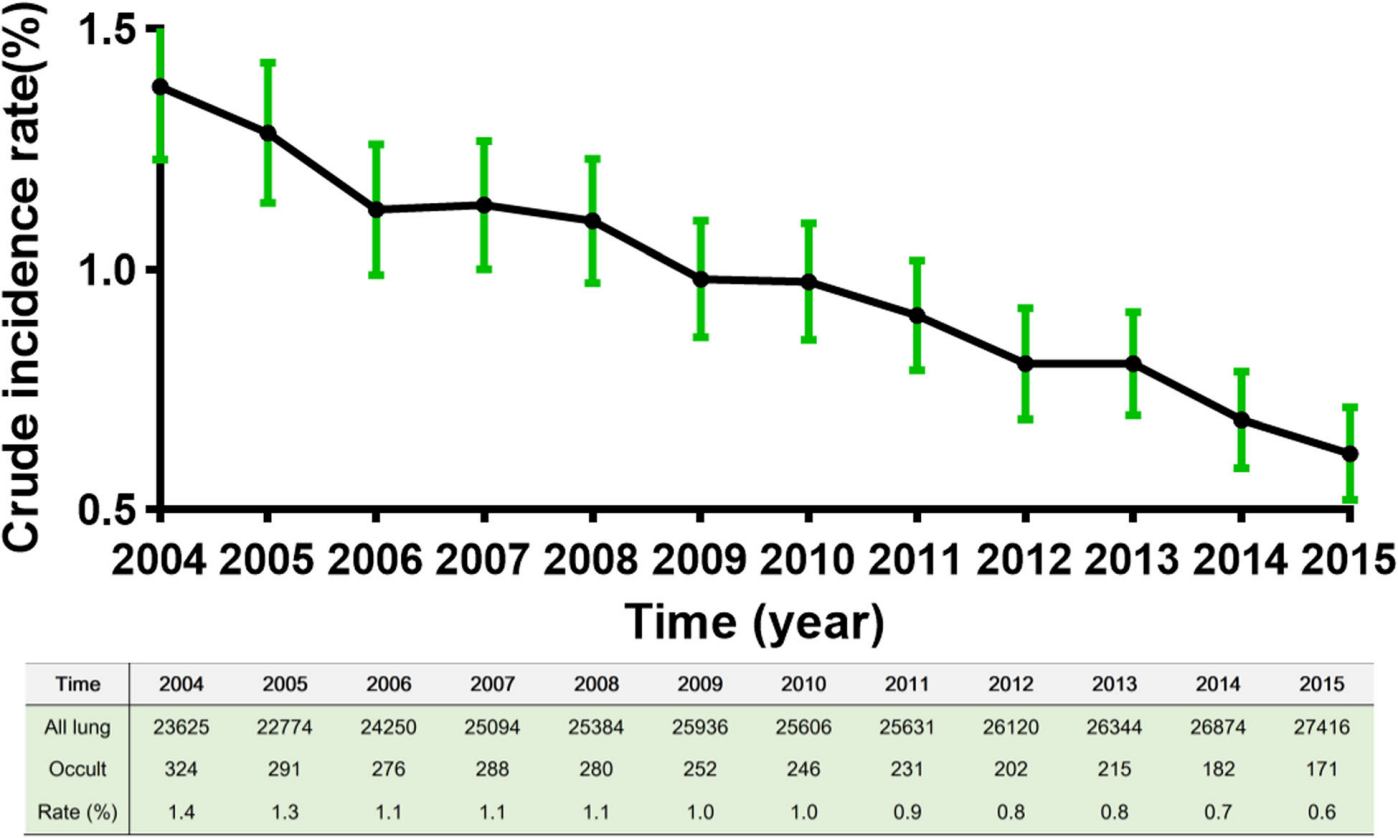
The crude incidence rate of occult lung cancer over time in the 305,054 lung cancer patients

In the all 305,054 patients, the crude incidence rate was 1.00 per 100 patients, and it was reduced between 2004 and 2015 [Fig. 2, 1.4 (95% CI 1.22–1.52) per 100 persons in 2004; 0.6 (95% CI 0.53–0.72) per 100 persons in 2015; Table 3, adjusted RR = 0.437, 95% CI 0.363–0.527]. The results of Linear regression revealed that trends about crude incidence rate of occult lung cancer was decreased over time (R = ˗0.023, *p* < 0.001).

**Table 3:** The results of multivariable Logistic regression analyses

	Multivariable analysis		
	RR	95% CI	***P***-Value
Year at diagnosis			
2004	1	reference	
2005	0.927	0.790–1.087	0.349
2006	0.828	0.705–0.974	0.022
2007	0.828	0.706–0.972	0.021
2008	0.792	0.674–0.930	0.005
2009	0.696	0.589–0.821	< 0.001
2010	0.685	0.580–0.809	< 0.001
2011	0.643	0.543–0.762	< 0.001
2012	0.549	0.460–0.655	< 0.001
2013	0.578	0.486–0.687	< 0.001
2014	0.479	0.399–0.575	< 0.001
2015	0.437	0.363–0.527	< 0.001
Sex			
Male	1	reference	
Female	0.988	0.919–1.063	0.752
Age (continuous)	1.035	1.031–1.038	< 0.001
Race			
Non-Hispanic whites	1	reference	
Non-Hispanic others	0.925	0.842–1.016	0.102

*RR* risk ratio, *CI* confidence interval

Logistic regression’s method was Enter selection

The results of multivariable analysis were adjusted for other confounding factors, such as sex, age, and race/ ethnicity

### Survival analysis of T classification

**Fig. 3:**
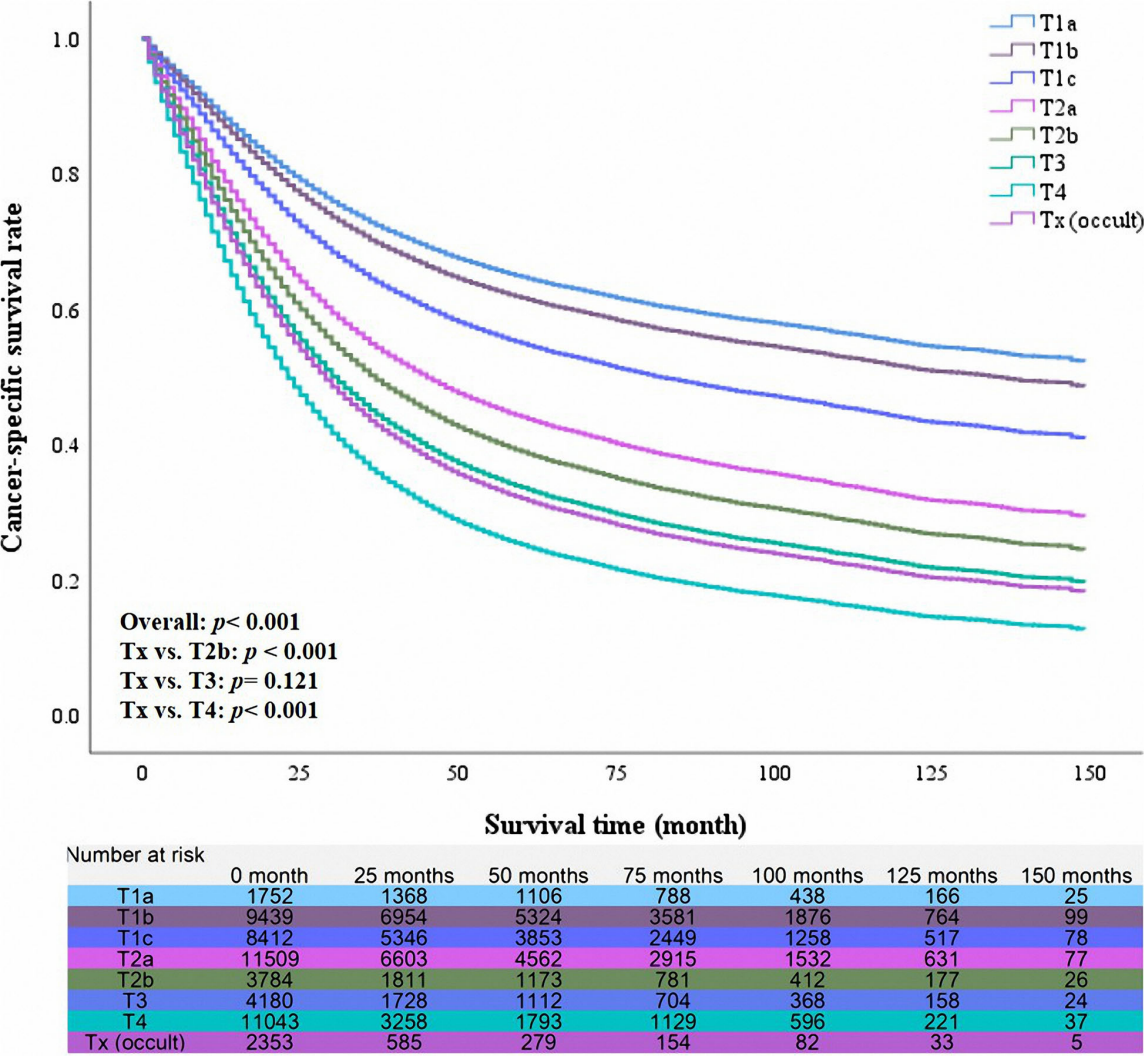
The adjusted survival curves of different T classifications

The median survival time of all 52,472 patients was 27 months (range from 1 month to 155 months). Besides, the 1-year, 3-year and 5-year cancer-specific survival rate of this cohort were 62, 49, and 44%, respectively. The unadjusted 5-year cancer-specific survival rate was the best in the patients with T1a (75%) and the worst in the patients with Tx (15%). The median survival time was 13 months (95% CI 12.10–13.90 months) in the patients with Tx, which indicated the rate of death events had exceeded 50%. We also found that the classification of Tx was the riskiest factor for the prognoses (Table 4, unadjusted HR =6.339, *p* < 0.001). However, the results were not inconsistent after multivariable Cox regression analysis. We used multivariable Cox regression analysis to identify the prognostic role of Tx (occult lung cancer) in the different T classifications (Table 4). After adjusting for other confounders, patients with Tx had a poorer prognosis than patients of T2b (adjusted HR 1.186, *p* < 0.001), nevertheless better long-term survival outcomes than patients with T4 (adjusted HR 0.845, *p* < 0.001). Besides, the prognosis for patients of Tx was not statistically different from that of T3 patients (*p* = 0.121). The adjusted survival curves also presented similar results (Fig. 3).

**Table 4:** Univariable and multivariable Cox regression analyses for prognostic factors

	N	5-year CSS	Univariable analysis		Multivariable analysis		
			HR	***P***-Value	HR	95% CI	***P***-Value
T classification				< 0.001			< 0.001
T1a	1752	75%	1		1	reference	
T1b	9439	67%	1.230	< 0.001	1.113	1.010–1.227	0.030
T1c	8412	56%	1.788	< 0.001	1.379	1.252–1.519	< 0.001
T2a	11,509	47%	2.345	< 0.001	1.889	1.719–2.076	< 0.001
T2b	3784	37%	3.079	< 0.001	2.172	1.964–2.402	< 0.001
T3	4180	31%	3.759	< 0.001	2.510	2.273–2.772	< 0.001
T4	11,043	19%	5.555	< 0.001	3.178	2.892–3.493	< 0.001
Tx (occult)	2353	15%	6.344	< 0.001	2.624	2.365–2.910	< 0.001
Subgroup comparison							
Tx vs. T2b	–	–	2.020	< 0.001	1.186	1.104–1.273	< 0.001
Tx vs. T3	–	–	1.648	< 0.001	1.054	0.986–1.127	0.121
Tx vs. T4	–	–	1.127	< 0.001	0.845	0.801–0.891	< 0.001

*HR* hazard ratio, *CI* confidence interval

Cox regression’s method was Enter selection

The results of multivariable analysis were adjusted for other confounding factors, such as sex, age, tumor differentiation, radiotherapy, chemotherapy, surgical treatment, histological subtypes, marital status, tumor location and race/ ethnicity

### Prognostic analysis for occult lung cancer

There were 2353 occult lung cancer patients for survival analyses, of which baseline characteristics were shown in Table 5. In this cohort, there were 2206 death events in the 2353 occult lung cancers. Female patients showed a better survival than male patients (Table 6, adjusted HR = 0.796, 95%CI 0.726–0.876, *p* < 0.001). Besides, the prognosis in patients with age > 72 years was poorer than younger patients (adjusted HR = 1.183, 95%CI 1.063–1.295). The number of adenocarcinomas was the most, which accounted for 33.0% (*N* = 776). Its long-term survival outcomes were better than squamous cell carcinomas (adjusted HR = 0.878, *p* = 0.042). Of note, 1162 patients didn’t receive any treatment. However, patients who underwent surgical resection or radiotherapy had improved survival benefits (Table 6). One-hundred and twenty-six patients underwent lobectomy, whose 5-year cancer-specific survival rate reached 47%. After adjusting for other confounders, we identified sex, tumor differentiation, tumor location, age, histological subtypes, radiotherapy, and surgical treatment as independent prognostic factors.

**Table 5:** Baseline characteristics in the cohort with occult lung cancer

Variables	All patients (***N*** = 2353)	Percentage (%)
Sex		
Male	1235	52.5
Female	1118	47.5
Tumor differentiation		
Well	186	7.9
Moderate	409	17.4
Poor	686	29.1
Unknown	1072	45.6
Tumor location		
Upper lobe	1096	46.6
Middle lobe	105	4.5
Lower lobe	668	28.4
Other location	105	4.5
Unknown	379	16.0
Age (year)		
≤ 72	1210	51.4
> 72	1143	48.6
Median (range)	72 (19–99)	
Histological subtypes		
Squamous cell carcinoma	726	30.9
Adenocarcinoma	776	33.0
Non-squamous and non-adenocarcinoma NSCLC	165	7.0
Small-cell carcinoma	300	12.7
Unknown non-sarcoma carcinoma	55	2.3
Unknown NSCLC	331	14.1
Chemotherapy		
No	1677	71.3
Yes	676	28.7
Radiotherapy		
No	1703	72.4
Yes	623	26.5
Unknown	27	1.1
Marital status		
Married	1092	46.4
Non-married	1116	47.4
Unknown	145	6.2
Race/ ethnicity		
Non-Hispanic whites	1928	81.9
Non-Hispanic other	425	18.1
Surgical treatment		
None	2077	88.3
Limited resection	77	3.3
Lobectomy	126	5.4
Pneumonectomy	7	0.3
Unknown surgical approach	4	0.2
Unknown	62	2.5

*NSCLC* non-small cell lung cancer

**Table 6:** Univariable and multivariable Cox proportional hazard regression analyses for prognostic factors in 2353 occult lung cancer patients

Variables	5-year CSS	Univariable analysis		Multivariable analysis		
		HR	***P***-Value	HR	95% CI	***P***-Value
Sex						
Male	14%	1		1	reference	
Female	16%	0.830	< 0.001	0.796	0.723–0.876	< 0.001
Tumor differentiation						
Well	18%	1		1	reference	
Moderate	17%	1.062	0.564	0.654	0.769–1.179	0.654
Poor	13%	1.373	0.001	1.214	1.003–1.506	0.046
Unknown	15%	1.152	0.131	1.038	0.860–1.264	0.669
Tumor location						
Upper lobe	17%	1		1	reference	
Middle lobe	9%	1.090	0.447	1.141	0.913–1.427	0.246
Lower lobe	14%	1.110	0.067	1.124	1.004–1.259	0.042
Other location	18%	1.115	0.354	1.036	0.822–1.305	0.767
Unknown	11%	1.291	< 0.001	1.241	1.084–1.421	0.002
Age (median, year)						
≤ 72	16%	1		1	reference	
> 72	14%	1.237	< 0.001	1.183	1.072–1.305	0.001
Histological subtypes						
Squamous cell carcinoma	13%	1		1	reference	
Adenocarcinoma	14%	0.890	0.05	0.878	0.775–0.995	0.042
Non-squamous and non-adenocarcinoma NSCLC	24%	0.680	< 0.001	0.754	0.609–0.933	0.010
Small-cell carcinoma	12%	0.992	0.920	0.958	0.812–1.131	0.616
Unknown non-sarcoma carcinoma	31%	0.647	0.022	0.558	0.380–0.820	0.003
Unknown NSCLC	15%	0.952	0.518	0.923	0.790–1.079	0.317
Chemotherapy						
No	17%	1		1	reference	
Yes	10%	0.926	0.132	0.946	0.846–1.059	0.338
Radiotherapy						
No	15%	1		1	reference	
Yes	16%	0.795	< 0.001	0.716	0.638–0.802	< 0.001
Unknown	12%	1.077	0.730	1.020	0.666–1.562	0.928
Marital status						
Non-married	14%	1				
Married	15%	0.990	0.837			
Unknown	17%	1.045	0.662			
Race/ ethnicity						
Non-Hispanic whites	15%	1		1	reference	
Non-Hispanic other	16%	0.995	0.938	1.063	0.941–1.202	0.323
Surgical treatment						
None	12%	1		1	reference	
Limited resection	30%	0.462	< 0.001	0.476	0.354–0.640	< 0.001
Lobectomy	47%	0.285	< 0.001	0.269	0.269–0.352	< 0.001
Pneumonectomy	13%	0.476	0.098	0.427	0.176–1.032	0.059
Unknown surgical approach	NA	0.506	0.238	0.544	0.175–1.695	0.294
Unknown	16%	0.972	0.847	0.899	0.673–1.203	0.475

*HR* hazard ratio, *CI* confidence interval, *NSCLC* non-small cell lung carcinoma

Cox regression’s method was Enter selection

## Discussion

In the present study, we used the data of 305,054 patients (including 2958occult lung cancer patients) to perform incidence-rate analysis. The results revealed that the crude incidence rate of occult lung cancer was 1.00 per 100 patients, and the incidence-rate trend over time was likely to be reduced between 2004 and 2015. Next, data on 52,472 eligible patients were analyzed by Cox regression analysis including univariable and multivariable analyses. Those patients included 2353 cases of occult lung cancer. According to the results, we found that occult lung cancer patients didn’t have satisfactory survival outcomes. The prognosis of occult lung cancer was between T2b’s and T4’s. Besides, there was no significant difference in the prognosis of patients with T3 classification or occult lung cancer. After adjusting for other confounders, the female, age ≤ 72, well differentiation, adenocarcinoma, radiotherapy, and surgical resection were considered as independent protective prognostic factors for 2353 occult lung cancer patients. Therefore, we suggested that surgery might be an appropriate option for occult lung cancer.

The incidence rate of occult lung cancer varied to a certain extent in the different populations. Previous studies and case reports found that occult lung cancer was usually accompanied by symptoms, metastatic diseases or other internal-medicine diseases when it was detected [6, 13–16]. *Yoel Siegel* et al. described a case report that occult lung cancer could mimic pneumonia and a pulmonary embolus by occluding a pulmonary vein [7]. A case by *William Carrera* et al. presented that occult small cell lung cancer might have a relation with occurring of retinopathy with chorioretinitis and optic neuritis [8]. Besides, *Hui Mai* et al. performed a study about characteristics of occult lung cancer-associated ischemic stroke, and suggested that occult cancer should be considered in the setting of multiple and recurrent embolic strokes within the short term in the absence of conventional stroke etiologies [9]. The above cases and study showed that occult lung cancer might be accompanied by different clinical symptoms. However, clinicians tend to pay more attention to their specialties, thus the diagnosis of the occult lung cancer becomes more complicated. Therefore, the research on the incidence rate of this disease may provide clinicians with some references for disease diagnosis and treatment.

Because malignant tumors may cause the blood to hypercoagulable state, which leads to the occurrence of thrombosis [17], the previous researchers began to investigate the incidence of occult lung cancer in stroke patients. *Alejandro Daniel Babore* et al. analyzed data of over 800,000 patients, and uncovered that the prevalence of occult lung cancer was 5.3 per 1000 patients in the stroke patients, and 2.6 per 1000 patients in the control group [6]. The sample size of their study was large, therefore, the results had clinical reference value. However, the results of the present study revealed that crude incidence rate of occult lung cancer was 10.0 per 1000 patients, which was much higher than the findings from above study. This difference in the incidence-rate results between above two studies was likely to be due to different selected cohorts. Our study cohort focused on lung cancer, which led to a higher incidence rate of occult lung cancer in the present study. However, the study by *Alejandro Daniel Babore* et al. majorly compared the incidence rate of occult lung cancer in stoke patients with that in general patients. Besides, they tried to explore the factors which might have effect on incidence rate. Though, this present study paid more attention to the incidence rate of occult lung cancer in entire lung cancer cohort, and illustrated that the incidence rate over time was reduced between 2004 and 2015. The reason why general trend over time was declined might be the popularization of computed tomography screening and the promotion of bronchoscopy [4, 18, 19].

The present study found that the prognosis of occult lung cancer patients was poorer than that in patients with T2 disease. Those patients might have occult metastasis of lymph node or another organ, which leads to a poor prognosis. Of note, timely therapy could improve the long-term survival in the occult lung cancer. Patients who underwent surgical resection had better cancer-specific survival than patients who didn’t receive surgical treatment. And, the best survival benefit was derived from lobectomy. *Joel J. Bechtel* et al. and *Cortese DA* et al. had similar findings in their research [20, 21]. They suggested that 5-year survival rate was 74 and 90% in patients with cure resection, respectively. However, the sample size was relatively small in their research [20, 21]. For example, in the study by *Joel J. Bechtel* et al., only 27 of the 51 patients they enrolled underwent surgical resection. Similarly, there were only 54 patients underwent operation in the study by *Cortese DA* et al. The sample size of the present study was different from above mentioned studies causing the difference of 5-year survival rate followed surgical resection. Besides, radiotherapy was proven to have survival benefit in the 71-case study by *M Saito* et al [22]. In the present study, compared with patients who didn’t receive radiotherapy, cases with radiotherapy had a better survival. These findings confirmed the results from previous study.

This study has several limitations. First, some important information (such as the invasion depth of tumor in the endobronchial wall) wasn’t detailed, as we couldn’t obtain the results of bronchoscopy and radiology in the SEER database. Thus, we did not further categorize the Tx classification. Second, cases with second primary lung cancer were excluded from the study. However, the incidence rate of occult lung cancer might be much higher in the cohort of second primary lung cancer. Therefore, the use of those findings was limited to patients with primary lung cancer. Third, because the data on histological subtypes were not detailed enough, unknown non-sarcoma cancer and unknown non-small cell carcinoma couldn’t be subdivided. Finally, this study belonged to retrospective study. Therefore, more studies are necessary to further explore the incidence rate and prognosis in patients with occult lung cancer.

## Conclusions

Occult lung cancer was uncommon. However, the cancer-specific survival of occult lung cancer was poor, therefore, we should put the assessment of its prognoses on the agenda. Timely surgical treatment and radiotherapy could improve survival outcomes. Besides, we still need more research to confirm those findings.

## Data Availability

Any researchers interested in this study could contact corresponding author for requiring data.

## Abbreviations

NSCLC: Non-small cell lung cancer
SCLC: Small cell lung cancer
TNM: Tumor-lymph node-metastasis
SEER: Surveillance, Epidemiology, and End Results
RR: Risk ratio
HR: Hazard ratio
CI: Confidence interval
